# Establishment of a modified CRISPR/Cas9 system with increased mutagenesis frequency using the translational enhancer dMac3 and multiple guide RNAs in potato

**DOI:** 10.1038/s41598-018-32049-2

**Published:** 2018-09-13

**Authors:** Hiroaki Kusano, Mariko Ohnuma, Hiromi Mutsuro-Aoki, Takahiro Asahi, Dai Ichinosawa, Hitomi Onodera, Kenji Asano, Takahiro Noda, Takaaki Horie, Kou Fukumoto, Miho Kihira, Hiroshi Teramura, Kazufumi Yazaki, Naoyuki Umemoto, Toshiya Muranaka, Hiroaki Shimada

**Affiliations:** 1 Department of Biological Science and Technology, Tokyo University of Science, Katsushika, Tokyo 125-8585 Japan; 2 Division of Field Crop Research and Development, Hokkaido Agricultural Research Center, NARO, 9-4 Shinsei-minami, Memuro, Kasai, Hokkaido 082-0081 Japan; 3 Laboratory of Plant Gene Expression, Research Institute for Sustainable Humanosphere, Kyoto University, Gokasho, Uji 611-0011 Japan; 4 Department of Biotechnology, Graduate School of Engineering, Osaka University, Suita, Osaka 565-0871 Japan; 5 RIKEN Center for Sustainable Resource Science, Tsurumi-ku, Yokohama, Kanagawa 230-0045 Japan

## Abstract

CRISPR/Cas9 is a programmable nuclease composed of the Cas9 protein and a guide RNA (gRNA) molecule. To create a mutant potato, a powerful genome-editing system was required because potato has a tetraploid genome. The translational enhancer dMac3, consisting of a portion of the *OsMac3* mRNA 5′-untranslated region, greatly enhanced the production of the protein encoded in the downstream ORF. To enrich the amount of Cas9, we applied the dMac3 translational enhancer to the Cas9 expression system with multiple gRNA genes. CRISPR/Cas9 systems targeting the potato *granule-bound starch synthase I* (*GBSSI*) gene examined the frequency of mutant alleles in transgenic potato plants. The efficiency of the targeted mutagenesis strongly increased when the dMac3-installed Cas9 was used. In this case, the ratio of transformants containing four mutant alleles reached approximately 25% when estimated by CAPS analysis. The mutants that exhibited targeted mutagenesis in the *GBSSI* gene showed characteristics of low amylose starch in their tubers. This result suggests that our system may facilitate genome-editing events in polyploid plants.

## Introduction

The clustered regulatory interspaced short palindromic repeats (CRISPR)/CRISPR-associated (Cas) system, which originated as a bacterial adaptive immune system, is a programmable nuclease composed of a Cas9 protein and a guide RNA (gRNA) molecule^1^. Currently, numerous applications have been reported for CRISPR/Cas9-mediated genome engineering in various organisms. In this system, Cas9 mediates a double-strand break of DNA at the target site, and the injured DNA is repaired by the non-homologous end joining process, which can result in a frameshift mutation and often results in the creation of a genetic knockout^2,3^.

Mutation frequencies differ depending on the Cas9 expression cassette used, and using an expression vector harboring the best combined Cas9/gRNA expression cassette results in an improved frequency of targeted mutagenesis^4^. In addition to the high-level expression of the *CAS9* gene, efficient translation of the mRNA may also allow the generation of a large amounts of Cas9 protein, resulting in increased efficiency toward the targeted mutagenesis. The 5′-untranslated region (5′-UTR) of certain mRNAs is known to act as a translation enhancer, largely increasing the production of the protein encoded by the downstream ORF^5^. The 5′-UTR from the alcohol dehydrogenase (ADH) gene has been applied as a translational enhancer in various studies focused on plant gene expression^6^. We have found long 5′-UTRs in the rice *OsMac1*, *OsMac2*, and *OsMac3* genes that show strong translational enhancer activity. The 5′-UTRs lead to a 10-fold or greater increase in the translational efficiency of the downstream ORFs^7,8^.

Potato, *Solanum tuberosum* L., has a tetraploid genome and commonly shows a vegetative reproduction manner. This finding implies that there are large difficulties in obtaining homozygous mutants in which all of the target genes have been altered. Many challenges have to be overcome to establish potato mutants through one-step engineering processes using programmable nucleases. The first successful case of targeted mutagenesis created a mutant for which *SSR2* gene was knocked out by TALENs^9^. To date, genome engineering techniques have been applied to create various gene mutants^10–15^.

The *granule bound starch synthase I* (*GBSSI*) gene is typically a model target for genetic engineering in potato. A *GBSSI*-deficient mutant shows amylose-free starch in potato tubers. This phenotype is easily detected by visualization using the iodide staining method, as the starch properties are largely altered^16^. Amylose-free starch is commercially valuable and used for food industries and manufacturing paper^17^. An established *GBSSI*-mutant has been obtained by random mutagenesis^18^. Additionally, amylose-free potato transgenic plants have also been created using antisense genes^19^, RNA interference^20^, and the CRISPR/Cas9 system^14^. We also have generated mutant potato alleles using *GBSSI*-targeted TALENs^21^.

In this study, we established an improved CRISPR/Cas9 vector system that contained a translational enhancer sequence derived from *OsMac3* for the efficient translation of Cas9. We examined the effect of multiple gRNAs for the potato *GBSSI* gene as the target gene. In this study, we describe the evaluation of this system and show the results from genome editing on the *GBSSI* gene. We also discuss the usefulness of our translational enhancer for the activation of Cas9 translation and demonstrate its contribution to the efficiency of targeted mutagenesis.

## Results

### Construction of a novel vector system containing the dMac3 translational enhancer

**Figure 1:**
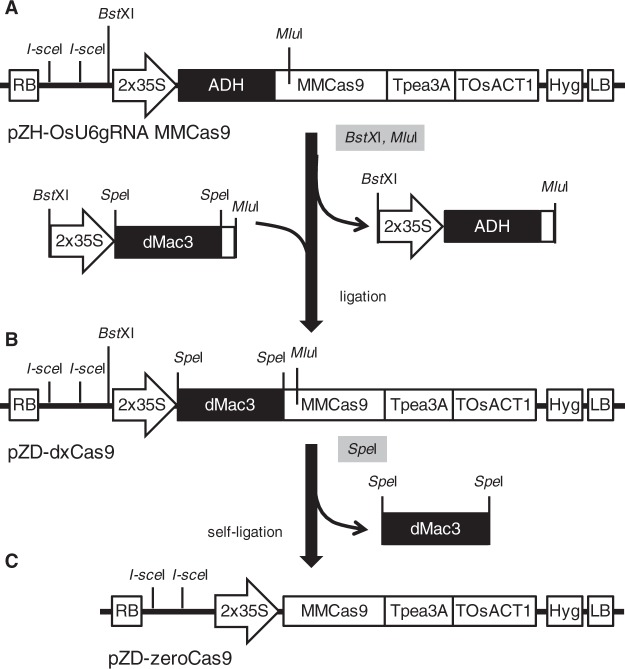
Construction of the improved CRISPR/Cas9 vectors used in this study. Structures of the regions in pZH-OsU7gRNA MMCas9 (**A**), pZD-dxCas9 (**B**), and pZD-zeroCas9 (**C**) are shown. Each figure indicates the region between the T-DNA right border (RB) and left border (LB) that contain the Cas9 gene and two *I-Sce*I sites for gRNA gene introduction. ADH: the ADH enhancer sequence; dMac3: a translational enhancer dMac3; MMCas9: MMCas9 coding region; 2 × 35 S: double CaMV 35 S promoter; Tpea3A: pea3A terminator; TOsACT1: rice actin1 terminator; Hyg: hygromycin resistant gene; RB: T-DNA right border; and LB: T-DNA left border.

We have reported that the *OsMac3* mRNA 5′-UTR acts as a translational enhancer that significantly promotes the translational efficiency of the downstream ORF^8^. We found that the 3′-portion of this sequence consisting of 161 nucleotides, named dMac3, showed sufficient activity as a translational enhancer. Using this sequence, we constructed a novel CRISPR/Cas9 vector system based on the established CRISPR/Cas9 system plasmid pZH_OsU6gRNA_MMCas9, which contains the Cas9 ORF following an ADH enhancer sequence^4^. The ADH sequence was replaced by dMac3, producing a pZD_dxCas9 plasmid that contains the Cas9 ORF following dMac3 (Fig. 1). Additionally, by removing the dMac3 region from this plasmid, we prepared the pZD-zeroCas9 plasmid that had no enhancer sequence linked to the Cas9 ORF (Fig. 1).

### Evaluation of CRISPR/Cas9 vectors containing a translational enhancer sequence

**Figure 2:**
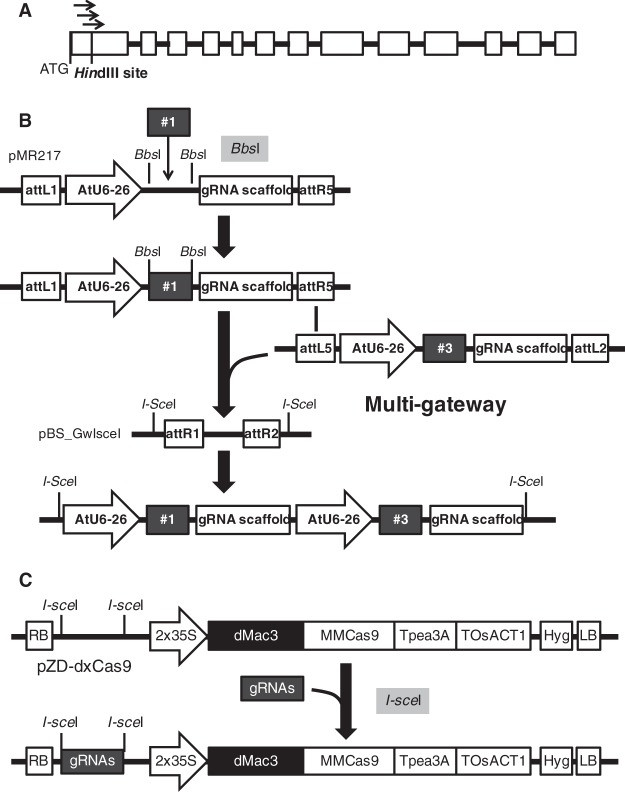
Construction of the CRISPR/Cas9 vector. (**A**) Structure of the potato *GBSSI* gene and the target site of guide RNAs. Boxes indicate the region for exons. Arrows indicate the locations of the guide RNAs. (**B**) Construction of the multiple gRNA genes. This figure shows a scheme of construction on the case using two gRNAs, gRNA #1 and #3 as the representative guide RNAs. DNA fragments corresponding to the target sequences were chemically synthesized and each of them was inserted between the AtU6 promoter and gRNA scaffold in a pMR vector. The resultant gRNA genes were introduced into pBS_GwIsceI using the multi-Gateway method. AtU6-26: *Arabidopsis thaliana* U6-26 promoter; gRNA scaffold: region for the gRNA scaffold; and attL1 and attL5 and attR1 and attR2: representative att regions for Gateway recombination sites. (**C**) Construction of the CRISPR/Cas9 vector plasmids. This figure shows the CRISPR/Cas9 vector plasmid, which contains an appropriate gRNA gene and the Cas9 gene with dMac3. gRNAs: representative gRNA genes. Other elements are cited from those in Fig. 1.

**Figure 3:**
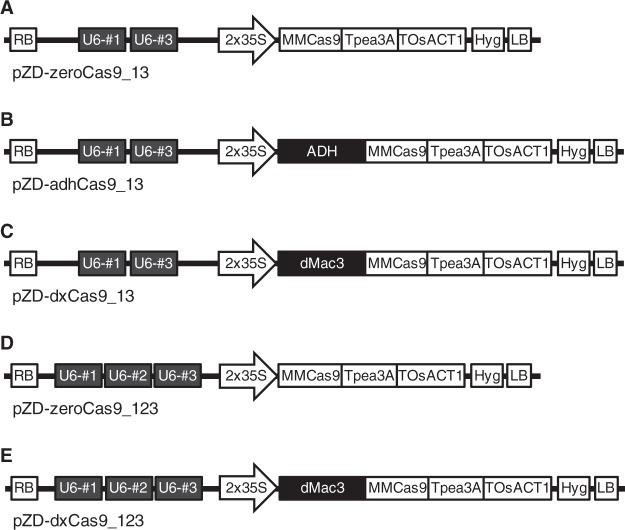
Structure of the CRISPR/Cas9 vectors used in this study. (**A**–**C**) Indicate the structure of CRISPR/Cas9s containing Cas9 with no enhancer sequence, the ADH enhancer, and dMac3, respectively, following two gRNA genes (gRNA #1 and gRNA #3). (**D**,**E**) Show vectors that containing three gRNA genes (gRNA #1, gRNA #2, and gRNA #3) followed by Cas9 with no enhancer sequence and dMac3, respectively. U6-#1, U6-#2, and U6-#3: gRNA genes for gRNA #1, #2, and #3, respectively. Other elements cite Figs 1 and 2.

We first evaluated the effects of the dMac3 and ADH translational enhancer sequences for the efficient translation of Cas9. To generate the CRISPR gRNAs, we selected three regions corresponding to sequences within the first exon in the potato *GBSSI* gene (Fig. 2A). These target sequences were placed between the AtU6-26 promoter and the chimeric single-guide RNA (gRNA) scaffold (Fig. 2B)^22,23^. The resultant gRNA genes were introduced into the Cas9 vector, resulting in the formation of CRISPR/Cas9 vectors containing tandem-located gRNA genes (Fig. 2C). As control plasmids, we prepared tandem-located gRNA genes associated with the Cas9 gene following the ADH enhancer, as well as plasmids with and without any translational enhancer (Fig. 3).

Potato transformants were created using the resultant plasmids. Potato has a tetraploid genome; thus, genome-editing events may occur on each of four alleles. We detected the mutant alleles in these transformants using a conventional Cleaved-Amplified-Polymorphic-Sequence (CAPS) analysis (Supplemental Fig. S1). The frequency of generated mutations was determined using a *Hin*dIII-digested/undigested PCR-amplified fragment containing the target sites, as there was a *Hin*dIII recognition site in the region corresponding to the gRNA target sequence.

**Figure 4:**
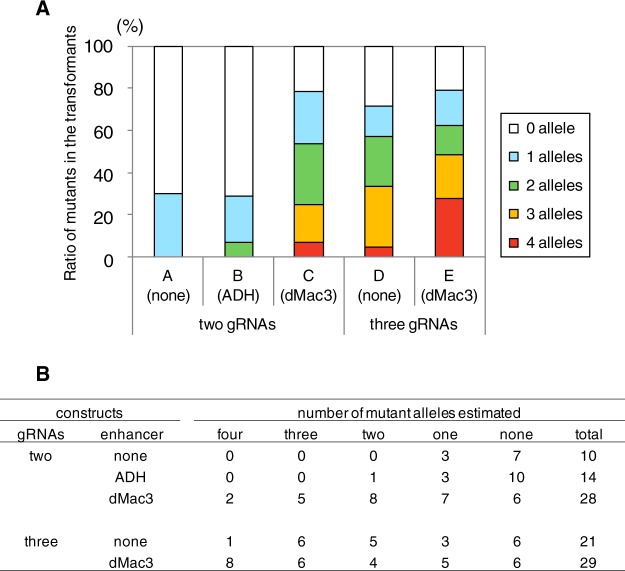
Determination of mutant alleles contained in the potato transformants. (**A**) Graphical representation on the ratio of mutants among the transformants shown in panel B. Numbers of mutant alleles are estimated by the difference in the CAPS analysis. Transformants are classified into 5 groups based on the estimated numbers of mutant alleles, which are indicated by colorimetric representation. A, B, and C indicate the results of target mutagenesis using CRISPR/Cas9 vectors that contain two gRNA genes with no enhancer sequence, the ADH enhancer, and dMac3, respectively. D and E indicate that results of target mutagenesis containing three gRNA genes with no enhancer sequence and dMac3, respectively. (**B**) The number of mutant transformants induced by each CRISPR/Cas9 system. Mutants are classified into 5 groups based on the number of mutant alleles estimated.

We determined the ratio of the targeted mutagenesis that occurred depending on each of the enhancer sequences when they were placed preceding the Cas9 ORFs in the CRISPR/Cas9 vectors containing two guide RNA genes. The number of mutant alleles in the transformants significantly increased when they contained the Cas9 genes with the ADH and dMac3 enhancer sequences compared to those generated without any enhancer sequences. Between the dMac3 and ADH enhancers, the transformants harboring the gene employing dMac3 showed a higher frequency of mutations (Fig. 4A). In this case, mutant alleles were detected in 22 of 28 (79%) transformants containing dMac3, whereas the mutant ratios were 3 of 10 (30%) and 4 of 14 (29%) in the transformants containing the ADH enhancer and no enhancer sequence, respectively (Fig. 4B). Mutation of multiple *GBSSI* gene alleles occurred in 15 of 28 (54%) transformants containing dMac3, whereas mutation of multiple alleles was only detected in 1 of 14 (7%) transformants containing the ADH enhancer sequence and in 0 of 10 transformants generated using CRISPR/Cas9 without any enhancer sequence (Fig. 4B). These results suggested that ratio of the mutations was strongly increased in the transformants harboring the dMac3 enhancer in the vector.

### Evaluation of multiple guide RNA genes in the CRISPR/Cas9 vector

We next analyzed the effects of the gRNA gene dosage introduced for the generation of *GBSSI* gene mutations. When three guide RNA genes were introduced into the CRISPR/Cas9 vectors (Fig. 3), the targeted mutation ratios increased in the transformants compared to those containing two guide RNA genes (Fig. 4A). Among them, three gRNAs with the CRISPR/Cas9 vector containing dMac3 showed the highest efficiency for targeted mutagenesis. In this case, 23 of 29 (75%) transformants had mutant *GBSSI* genes, with 8 transformants (28%) showing the mutation at all four alleles (Fig. 4B). In the transformants containing three gRNAs with no enhancer sequence, 15 of 21 transformants (71%) were mutants. Multiple mutant alleles were found in 12 transformants, although only one transformant (5%) showed the mutation of all four alleles (Fig. 4B). These results indicated that a higher targeted mutagenesis efficiency was generated using the CRISPR/Cas9 vector containing three gRNAs compared to those with two gRNAs.

### Sequence analysis of the target sites in the GBSSI gene mutant alleles

**Figure 5:**
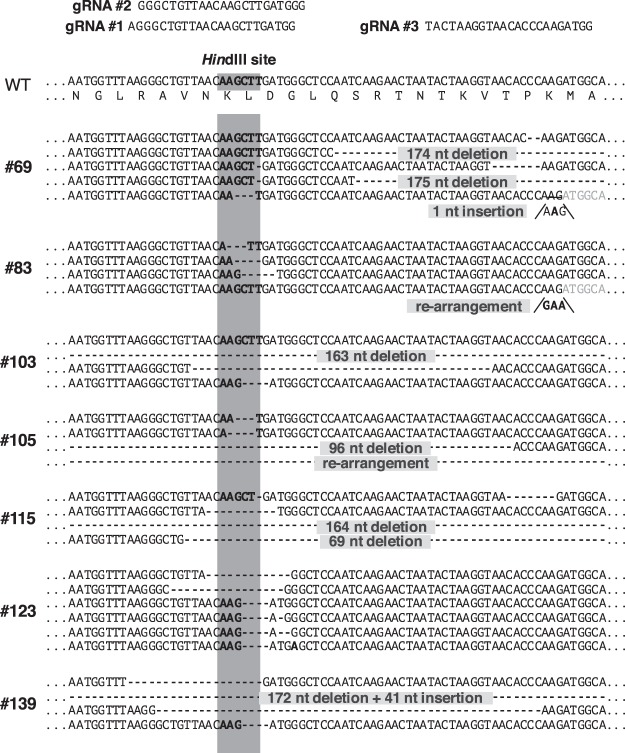
Nucleotide sequences around the target site in the potato *GBSSI* gene from the generated representative mutants. The corresponding wild-type nucleotide sequence (WT) is shown in the upper portion of the figure. The nucleotide sequences of the gRNAs (#1, #2, and #3) are indicated above. The region corresponding to the *Hin*dIII site is shaded and indicated by bold letters. Numbers with the prefix # indicate the individual transformants. When multiple DNA sequences were detected, they were aligned. Gaps indicate a nucleotide deletion in the mutants. Nucleotide insertion and replacement is shown as nucleotides on the appended line. Large numbers of nucleotide deletions and insertions are noted on the lines.

We determined the nucleotide sequences of the target regions in the *GBSSI* gene from the representative mutant plants. To identify the nucleotide sequences of the four alleles in the wild-type *GBSSI* genes, the target regions were PCR-amplified, subcloned, and used to determine the nucleotide sequences. Among 20 clones, we detected several nucleotide differences, which were considered to exhibit polymorphism. However, as far as we could determine, all of the clones showed the identical nucleotide sequence in the region corresponding to the guide RNAs (Fig. 5).

We next analyzed the nucleotide sequences of the representative mutants containing four or three mutant alleles. In this region, there were polymorphic DNA sequences around the mutant target regions. We detected four different nucleotide sequences in the transformants, #83, #105, #115, and #139, which were suggested to contain four mutant alleles (Fig. 5). Transformant #105 exhibited mutant alleles that contained nucleotide deletions of 3, 4, and 96 nt and a rearranged nucleotide sequence with both a 245-nt deletion and a 1479-nt insertion in the target region (Supplemental Fig. S2). Transformants #83, #115, and #139 also showed four different nucleotide sequences. In the target region of transformant #83, we detected deletions of 3, 4, and 5 nt and a AG to GAA substitution (Fig. 5). Transformant #115 showed 8, 10, 69 and 164 nt deletions in the target region. Transformant #139 contained three alleles with 4, 19, and 54 nt deletions and an allele with 172 nt deletion along with a 41 nt insertion (Fig. 5). Transformant #103 contained a wild-type nucleotide sequence, suggesting that this transformant possessed three mutant alleles (Fig. 5). We did not detect the wild-type sequences in the other clones. Among transformants #69 and #123, we detected five polymorphic fragments (Fig. 5). This result suggested that these mutant plants might consist of chimeric cells with *GBSSI* genes that were independently modified. In some transformants, such as #83 and #105, we found that the mutant alleles were generated by the deletion of three nucleotides in the target region (Fig. 5). This showed that these mutant genes encoding the mutant GBSSI proteins lack one amino acid residue.

### Analysis of the changes in the amylose contents in the transformant tubers

**Figure 6:**
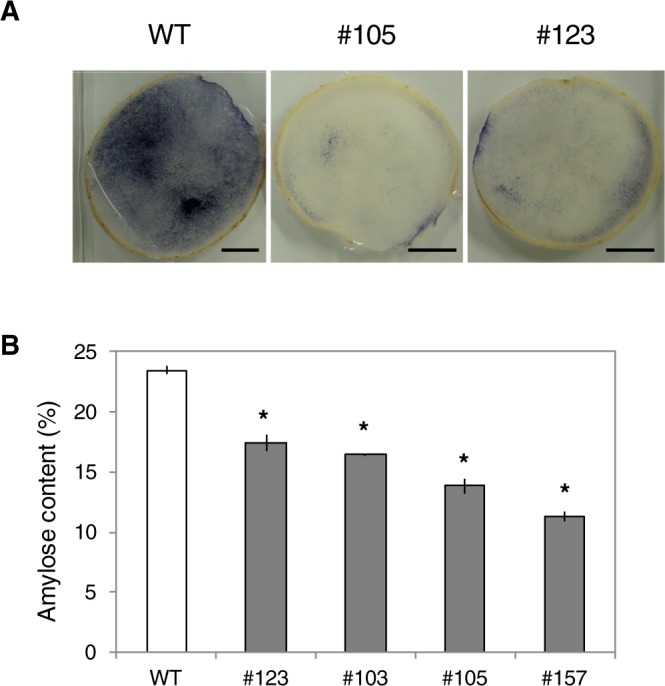
Analysis of tuber starch. (**A**) Iodine staining of potato tuber sections. WT, #105, and #123 show wild-type tuber sections and #105 and #123 show transformants. Bar = 2 cm. (**B**) Amylose content in the tuber starch from the four transformants and wild-type plants. Numbers with the prefix # indicate the individual transformants. Values show the average of triplicate measurements. An asterisk indicates statistical significance compared with the wild type (P < 0.05).

The mutant plants grew normally and yielded tubers. When the starch in the tubers from mutants #105 and #123 was analyzed by iodine-staining assay, these mutants showed low levels of staining compared with the wild-type plant (Fig. 6A). We analyzed the amylose contents in the tubers from the four representative mutants. As shown in Fig. 6B, significantly lower amylose contents were observed in the mutants compared with the wild-type plant.

## Discussion

We constructed a novel CRISPR/Cas9 vector system containing the dMac3 translational enhancer before the Cas9 nuclease gene along with multiple gRNA genes for target regions in the potato *GBSSI* gene (Fig. 2A). We evaluated the effect of the dMac3 translational enhancer for efficient mutagenesis of the target gene. We also determined the mutation efficiency using multiple guide RNA genes. It is expected that dMac3 may enhance the Cas9 translation, which could result in an elevated digestion efficiency at the target regions. Our results indicated that dMac3 was highly effective at increasing the mutagenesis efficiency. Multiple gRNA genes also increases the mutant allele ratio in the transformants (Fig. 4). We evaluated the mutation ratio using CAPS analysis. Among the three gRNAs, gRNA #3 was located at the region farthest from the *Hin*dIII site. This finding suggests that we might miss the detection of mutations at the gRNA #3 region. Indeed, we found mutations at the region corresponding to this gRNA in transformants #61, #65, #69, and #83 when the sequences were determined using nucleotide sequence analysis (Fig. 5).

In this experiment, we obtained mutant lines that had four mutant alleles in the *GBSSI* gene (Figs 4 and 5). Mutations in the *GBSSI* gene results in a reduction in the amylose content in the potato tuber storage starch. Mutants lacking GBSSI activities typically show the phenotype of production of amylose-free starch^14^. However, none of the mutants showed an amylose-free phenotype, though their amylose contents were significantly lower (Fig. 6). We detected various types of polymorphic DNA sequences that contained nucleotide rearrangements in the *GBSSI* gene target region. Because a number of transformants exhibited multiple mutant sequences in the target region, they were suggested to contain multiple mutant alleles. Among the mutant alleles, we detected a three-nucleotide deletion on one of the mutant alleles in transformants #83 and #105, though they were suggested to have four mutant alleles (Fig. 5). These deletions did not cause a frameshift leading to a premature stop codon. We presumed that the corresponding mutant proteins still maintained their functions, accounting for the existence of amylose in the mutant potato tuber starch. It was also notable that more than four different polymorphic DNA sequences were detected in mutants #69 and #123 (Fig. 5). This suggested that these mutants consisted of chimeric cells. Although we detected no more than five DNA sequences in other transformants, there might be other chimeric clones in the mutant plants.

Potato has an autotetraploid genome, and it is difficult to obtain progenies, which may lead to a mutant allele pyramid in the progenies. To establish a potato mutant, a powerful and innovative tool is required because site-specific mutations must be present on all four target gene alleles. In this study, we propose a novel plant genome-editing system containing a dMac3 translational enhancer and three gRNA genes. Using this system, we successfully generated potato mutants on multiple *GBSSI* gene alleles (Fig. 4). We observed that more than 28% of transformants containing four mutant alleles for the gene (Fig. 4). These observations indicate that our system may facilitate genome-editing events in polyploid plants, such as potato.

## Materials and Methods

### Plant materials and growth conditions

The *Solanum tuberosum* L. cultivar Sayaka was used. Tubers were harvested from wild-type and transformant potato, which were grown at 22 °C under 16 h-light and 8 h-dark conditions in a growth chamber.

### Transformation of potato plants

Potato was transformed using the *Agrobacterium*-mediated procedure using the *A*. *tumefaciens* EHA105 strain according to Yamada *et al*.^24^. Stem internodes were isolated from cultured potato plants grown for 3 weeks and cut into approximately 1 cm pieces. The pieces were infected with *Agrobacterium* harboring an appropriate plasmid. Callus induction and plant regeneration were performed on 3C5ZR agar plates^25^ supplied with 3 mg L^−1^ hygromycin for 2 months. Regenerated plants were used for the detection of the transgene by PCR using the Cas9-S (5′-GGCGTAAGAATAGAATCTGTTAT-3′) and Cas9-T (5′-GACAGCGCTATCAGATTTCCAA-3′) primers, which amplified part of the Cas9 gene sequence.

### Construction of the plasmids for CRISPR/Cas9

A fragment corresponding to dMac3 (Aoki *et al*. 2014) was chemically synthesized. The CRISPR/Cas9 vector pZH_OsU6gRNA_MMCas9^4^ was used to construct the CRISPR/Cas9 vectors in this work. The corresponding ADH enhancer region was removed from pZH_OsU6gRNA_MMCas9 and replaced with a fragment containing the dMac3 region. The resultant plasmid, named pZD-dxCas9, was used to construct the CRISPR/Cas9 vectors through the introduction of guide RNA genes. A CRISPR/Cas9 vector without any translational enhancer, pZD-zeroCas9, was created by removal of the Mac3 region from pZD-dxCas9. Construction of pZD-dxCas9, pZH_OsU6gRNA_MMCas9 and pZD-zeroCas9 is summarized in Fig. 1. Detailed procedures are described in the Supplemental methods.

For construction of the gRNA genes, the DNA fragments corresponding to the target sequences were chemically synthesized. *Bbs*I sites were included in the sequences on both sides of the fragments. The fragments were inserted into the *Bbs*I site in the appropriate guide RNA vector, such as pMR203, pMR204, pMR205, pMR217, and pMR218 (generously provided by M. Endo), and localized between the AtU6-26 promoter and the gRNA scaffold sequence. The resultant gRNA genes were utilized for the construction of the intermediate plasmids containing two or three gRNA genes. These plasmids were constructed by the introduction of gRNA genes into the pBS_GwIsceI plasmid using the multisite-Gateway system (Invitrogen, Carlsbad, CA, USA). The pBS_GwIsceI plasmid, which was derived from pGWB2 (accession No. AB289765), had a region for the Gateway attR1 and attR2 sites lying between the two *I-Sce*I sites. A detailed procedure for the construction of the pBS_GwIsceI is described in the Supplemental methods. The fragments corresponding to the tandem gRNA genes were prepared from the intermediate plasmids by *I-Sce*I digestion and inserted into the *I-Sce*I sites in the pZH_OsU6gRNA_MMCas9, pZD-dxCas9, pZD-zeroCas9 plasmids to generate the CRISPR/Cas9 vectors (Fig. 2).

### CAPS analysis

Genomic DNA was prepared from potato leaves using the RED Extract N-Amp Plant PCR kit (Sigma-Aldrich, St. Louis, USA). The 1.0-kb region in the potato *GBSSI* gene that contained the sequences of the target guide RNAs was amplified using the GBSS-G_XhoI (5′-CCCCTCGAGCTTGCCTACTGTAATCGGTGATAA-3′) and GBSS-N_BamHI (5′-CCCGGATCCCAAGCTGAACCTAAGTTCAT-3′) primers. The amplified fragment was digested with *Hin*dIII. The wild-type gene had a *Hin*dIII site in the corresponding region. Therefore, we judged this as a mutant allele when the amplified fragment was no longer digested by *Hin*dIII. The number of mutation alleles among the four genes were estimated by the ratio of undigested fragments to digested fragments. The amplified fragments were inserted into the pBluescriptII SK+ vector (acc. no. X52328) (Toyobo, Osaka, Japan) after digestion with *Xho*I and *Bam*HI and used for nucleotide sequence analysis.

### Iodine staining assay and analysis of amylose content in the tuber

Sliced sections of potato tuber were dipped into 0.2% potassium iodide-0.1% iodide solution. Photographs were taken after washing with water. The starch levels in the potato tuber were analyzed using powdered starch granules, which were obtained as described previously^26^. The amylose contents in the potato tubers were analyzed according to a previous paper^26^. The data were statistically analyzed using Student’s *t* test.

## Electronic supplementary material

Supplemental Materials
