# Supplementary material for: Establishment of a modified CRISPR/Cas9 system with increased mutagenesis frequency using the translational enhancer dMac3 and multiple guide RNAs in potato
Source: Sci Rep. 2018 Sep 13;8:13753. doi: 10.1038/s41598-018-32049-2 (PMC6137036; doi:10.1038/s41598-018-32049-2)
Supplement: Supplementary file 1 — Supplemental Materials [file 41598_2018_32049_MOESM1_ESM.pdf]

Establishment of a modified CRISPR/Cas9 system with increased mutagenesis frequency using the translational enhancer dMac3 and multiple guide RNAs in potato

Hiroaki Kusano[1,3], Mariko Ohnuma[1], Hiromi Mutsuro-Aoki[1], Takahiro Asahi[1], Dai Ichinosawa[1], Hitomi Onodera[1], Kenji Asano[2], Takahiro Noda[2], Takaaki Horie[1], Kou Fukumoto[1], Miho Kihira[1], Hiroshi Teramura[1], Kazufumi Yazaki[3], Naoyuki Umemoto[4], Toshiya Muranaka[5], Hiroaki Shimada[1]*

1. Department of Biological Science and Technology, Tokyo University of Science, Katsushika, Tokyo 125-8585, Japan
2. Division of Field Crop Research and Development, Hokkaido Agricultural Research Center, NARO, 9-4 Shinsei-minami, Memuro, Kasai, Hokkaido 082-0081, Japan
3. Laboratory of Plant Gene Expression, Research Institute for Sustainable Humanosphere, Kyoto University, Gokasho, Uji 611-0011, Japan
4. Department of Biotechnology, Graduate School of Engineering, Osaka University, Suita, Osaka 565-0871, Japan
5. RIKEN Center for Sustainable Resource Science, Tsurumi-ku, Yokohama, Kanagawa 230-0045, Japan

*Corresponding Author:
Hiroaki Shimada:
Tel: +81-3-5876-1469
E-mail: shimadah@rs.noda.tus.ac.jp

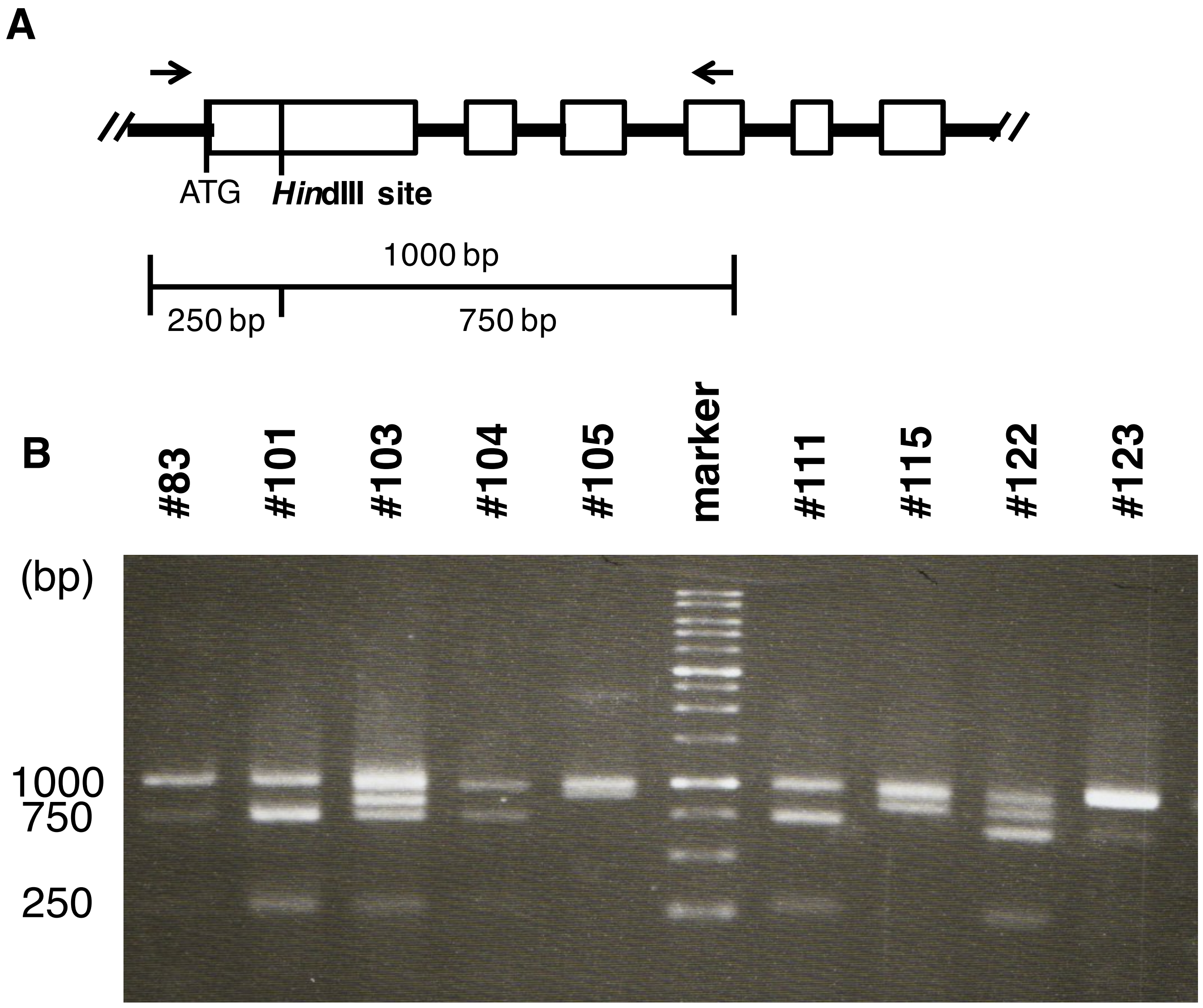

Supplemental Fig. S1. An example of the results of the CAPS assay on the potato transformants. (A) Location of the target region in the potato *GBSSI* gene. Boxes show the region for exons. ATG indicates the initiation codon. Arrows indicate the positions of PCR primers by which the region containing the target sequence was amplified. The wild-type gene contains a *Hin*dIII in the amplified region, and two fragments are generated by the CAPS analysis. (B) An example of gel image of the CAPS assay. Numbers with prefix # indicate the transformants. An approximately 1.0 kb fragment is amplified by PCR, and the wild-type fragment generates 0.25 kb and 0.75 kb fragments by *Hin*dIII-digestion. When the amplified fragment was undigested, we considered this locus had a mutation on the *Hin*dIII site. Numbers of mutant alleles were estimated by the ratio of undigested fragments to the digested ones.

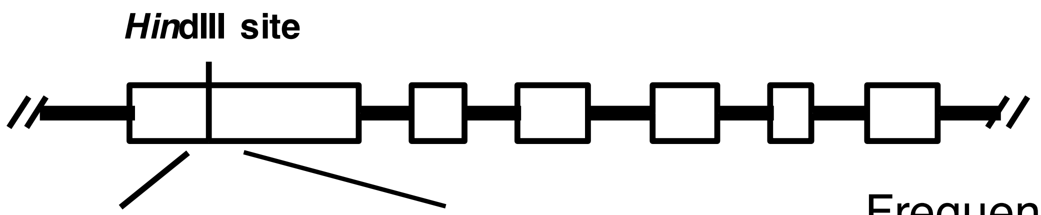

Frequency

A

3 nt deletion

GCTGTTAACAA---TGATGGGCTC **12/40**

-3 nt

TGGTTTAAGG**GCTGTTAACAATGATGGGCTC**CAATCAAGAAC

B

4 nt deletion

GCTGTTAACA----TGATGGGCTC **12/40**

-4 nt

ATGGTTTAAGG**GCTGTTAACATGATGGGCTC**CAATCAAGAAC

C

96 nt deletion

TCAACCTTGT---------------------ACCCAAGATG **15/40**

-96 nt

TAGACACCAAA**TCAACCTTGTACCCAAGATG**GCATCCAGAAC

D

245 nt deletion

GTCACCGATA---------------------CCTTGCAGTA **1/40**

+1479 nt insertion

```
AAATCAACCTTGTCACCGATAactcttcccgaggctctgataccagtttgtcgggaaaacacggacaaacacaaaaatatat
atggtaaaagtaatggaaataaaatgggaaataatgacaccaagaattttacgtggaaacccttctaaataagggaaaaact
acgggccaagaggagcaactgatattactatagtaaggaattttacactgtagtcacgagtacaatactcaaaatgactact
acacactcaaaaggaataacactcttttgatttccaccttactaaaatatcgctcacactctatttttcttcacagactatt
tttcttgtatagcctatggaaacctcacagctctctaaatatttactctctctgtgaattggtgtatttgggaatgagcaa
agggctctctatttatagaagaaatttgcagttctatacataatatgtcaaagttgtgccaccgaaagcattttttaattgct
gtgtttttcaacaattgctgctattgcaatttgccaacaattgacaattgcatgcagcaattgttgacactaaaaaaagaaa
agtgggactggaccccacaaatctccccctccagtcccatttactggaaggaggtatcttcatattctttagagagagctca
tacccacaagttctttgcataactcgaacttgtctttcagtatcgtcttggtcagcatatctgcaggattttcacttgtgtg
gatctttttgacgtgaaatgattcactttccacttcctcacgaatccaatgatatctgacgtcgatgtgttttgtccttgca
tggtacatggagttcttgctcaagtctattgcactctgactgtcacaatagacaatatactccatctgatgcaaaccaagct
cttgaagaaatcgcttgagccatatcatctccttgccggcttcagtagccgcaatatactcagcttcagttgtagatagtgc
aacacacttctgcaacttcgactgccatgatatagctccccccctgaaaaagtaaacaaatatccagtagtggattttctgtt
atcaaggtcacctgccatatcagcatctgtatagcccttcaagattggatctgatgctccaaaaacacaaacattcatctga
gcttcctctaagatacctgagtatccacttcacagcttcccaatgctcttttcccggattgtcgagaaatctgctaacaaca
ccaactgcgtgagcaatatcaggtctagtgcataccattgcatacattagacttccgacgacggaggaatatggaactttgg
ccatgctctctttttcctccctaactgtaggacacatctttttactcaacttcatatgacaagcaagaggtgtgctaacagg
cttagcattcttcatattgaagcgctctagtacacgttcaatgtacttcttctgtgacaaataaatcttcctttcatctctg

agacgagtaattctcatgcctaaaCCTTGCAGTAAGTCTTTCATT
```

Supplemental Fig. S2. Nucleotide sequences around the target site in the potato *GBSSI* gene of the transformant line #105. Panels A to D indicate the nucleotide sequences detected. Triangles indicate the position of mutation in the gene. The nucleotide sequence shown in panel D was generated by replacement of 245 nt of the *GBSSI* gene to an unidentified 1479 nt sequence. Frequency of appearance of each sequence was shown in the right side.

Supplemental Methods

Construction of guide RNA genes

In the case of the construction of the plasmid containing two guide RNA genes, #1 and #3, the corresponding guide RNAs were chemically synthesized, and were cloned into *Bbs*I sites of pMR217 and pMR218 (kindly provided by Dr. Masaki Endo), respectively (see Fig. 2). In the case of the construction of the plasmid containing three guide RNA genes, #1, #2, and #3, the chemically-synthesized corresponding fragments were cloned into *Bbs*I sites of pMR203, pMR204, and pMR205, respectively. The regions for AtU6-26 promoter, target sequence region, and gRNA scaffold region, which existed between *att* sequences, were introduced into *att* regions of pBS_GwIsceI. The resultant plasmid contained multiple guide RNA genes.

The plasmid pBS_GwIsceI was constructed as follows: The region for the Gateway *att*R1-*att*R2 unit was PCR-amplified from pGWB2 (accession No. AB289765) using primers, GWB2attR1-R_I-SceI_HindIII (5'-CCCAAGCTTTAGGGATAACAGGGTAATTTGGAGAGAACACGGGGGACTCTA-3') and GWB2attR2-F_I-SceI_SpeI (5'-CCCACTAGTTAGGGATAACAGGGTAATGGAAATTCGAGCTCTAAGCGCTGT-3'). The amplified fragment was digested by *Spe*I and *Hin*dIII, and inserted into the region between the *Spe*I and *Hin*dIII sites of pBluescriptII SK+ (Toyobo, Osaka, Japan). The resultant plasmid was named pBS_GwIsceI.

Legends to Supplemental Figures:

Fig. S1. An example of the results of the CAPS assay on the potato transformants. (A) Location of the target region in the potato *GBSSI* gene. Boxes show the region for exons. ATG indicates the initiation codon. Arrows indicate the positions of PCR primers by which the region containing the target sequence was amplified. The wild-type gene contains a *Hin*dIII in the amplified region, and two fragments are generated by the CAPS analysis. (B) An example of gel image of the CAPS assay. Numbers with prefix # indicate the transformants. An approximately 1.0 kb fragment is amplified by PCR, and the wild-type fragment generates 0.25 kb and 0.75 kb fragments by *Hin*dIII-digestion. When the amplified fragment was undigested, we considered this locus had a mutation on the *Hin*dIII site. Numbers of mutant alleles were estimated by the ratio of undigested fragments to the digested ones.

Fig. S2. Nucleotide sequences around the target site in the potato *GBSSI* gene of the transformant line #105. Panels A to D indicate the nucleotide sequences detected. Triangles indicate the position of mutation in the gene. The nucleotide sequence shown in panel D was generated by replacement of 245 nt of the *GBSSI* gene to an unidentified 1479 nt sequence. Frequency of appearance of each sequence was shown in the right side.
